# Pathological complete response of locally advanced colon cancer after preoperative radiotherapy: a case report and narrative review of the literature

**DOI:** 10.1186/s40792-018-0466-8

**Published:** 2018-06-15

**Authors:** Sho Sekiya, Kiyotaka Imamura, Shintaro Takeuchi, Koichi Teramura, Yusuke Watanabe, Eiji Tamoto, Minoru Takada, Yoshihiro Kinoshita, Yoshiyasu Anbo, Fumitaka Nakamura, Nobuichi Kashimura, Hiroko Noguchi, Katsutoshi Miura, Satoshi Hirano

**Affiliations:** 1 Department of Gastroenterological Surgery, Teine Keijinkai Hospital, 1-40, Maeda 1-12, Teine-ku, Sapporo, Hokkaido 006-8555 Japan; 2 Department of Pathology, Teine Keijinkai Hospital, 1-40, Maeda 1-12, Teine-ku, Sapporo, Hokkaido 006-8555 Japan; 3 Department of Radiology, Teine Keijinkai Hospital, 1-40, Maeda 1-12, Teine-ku, Sapporo, Hokkaido 006-8555 Japan; 4 Department of Gastroenterological Surgery II, Hokkaido University Faculty of Medicine, North-15, West-7, Kita-ku, Sapporo, Hokkaido 060-8648 Japan

**Keywords:** Colon cancer, Radiotherapy, Pathological complete response

## Abstract

**Background:**

The oncological effectiveness of preoperative radiotherapy for locally advanced colon cancer is unclear. We report a case of pathological complete response in a patient with locally advanced ascending colon cancer after preoperative radiotherapy following failure of chemotherapy.

**Case presentation:**

A 65-year-old Japanese woman presented with malaise and hematochezia. A computed tomography (CT) revealed a tumor in the ascending colon which seemed to infiltrate the adjacent structures. She was diagnosed with locally advanced ascending colon cancer stages T4b, N2a, M0, and IIIC. We selected modified FOLFOX6 with panitumumab as neoadjuvant chemotherapy. However, we discontinued the chemotherapy after the 8th cycle because of disease progression and severe adverse effects. The patient then underwent radiotherapy of 60 Gy in 30 fractions, resulting in significant tumor size reduction. One month after the radiotherapy, we performed a right hemicolectomy with multivisceral resection without complications. Histopathologically, we found no residual cancer cells in the resected specimen. The patient remains alive and has not required additional therapies for 24 months, as there are no signs of recurrence.

**Conclusions:**

The present case suggests that preoperative radiotherapy might be an effective treatment options for locally advanced colon cancer.

## Background

Locally advanced colon cancer is clinically defined as primary colon cancer with direct invasion to the adjacent structures or extensive regional lymph node involvement. Previous studies have shown low rates of pathological complete resection of locally advanced colon cancer and high incidence of postoperative morbidity and mortality because of the required multivisceral resection [1]. Five-year survival rates for patients with stage IIIB and IIIC colon cancer have been reported to be 46 and 28%, respectively [2]. Preoperative chemoradiotherapy is recommended for locally advanced rectal cancer since it can improve the oncological outcomes [3–5]. However, there is still uncertainty about the oncological effectiveness of preoperative radiotherapy for colon cancer. Herein, we report a case of locally advanced ascending colon cancer with pathological complete response after preoperative radiotherapy following failure of chemotherapy.

## Case presentation

**Fig. 1:**
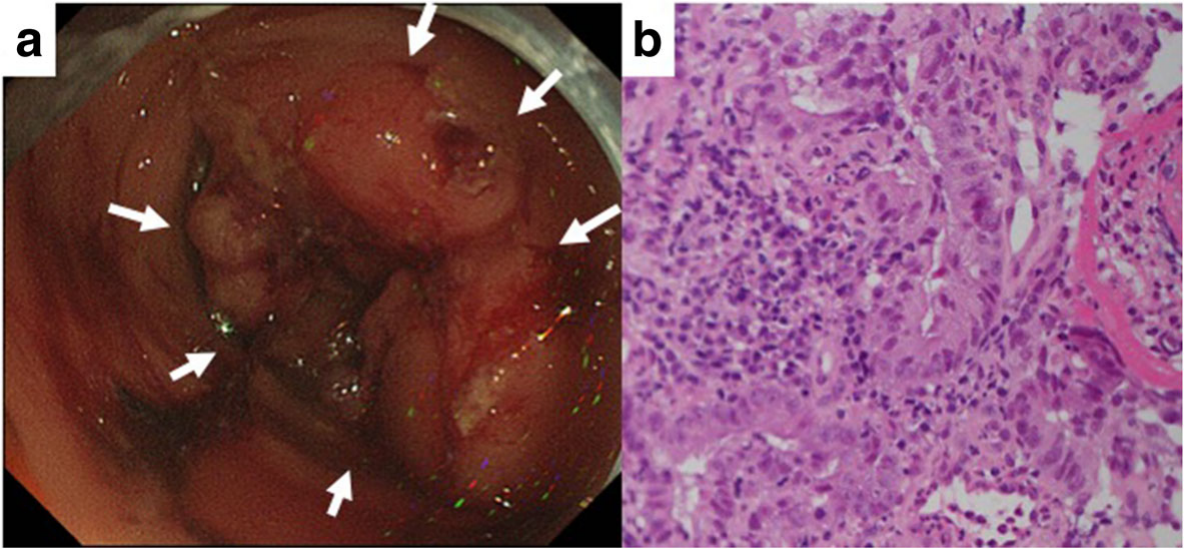
Images before the treatment. **a** Colonoscopy showing a circumferential neoplastic lesion in the ascending colon. **b** Biopsy revealed moderately differentiated tubular adenocarcinoma

**Fig. 2:**
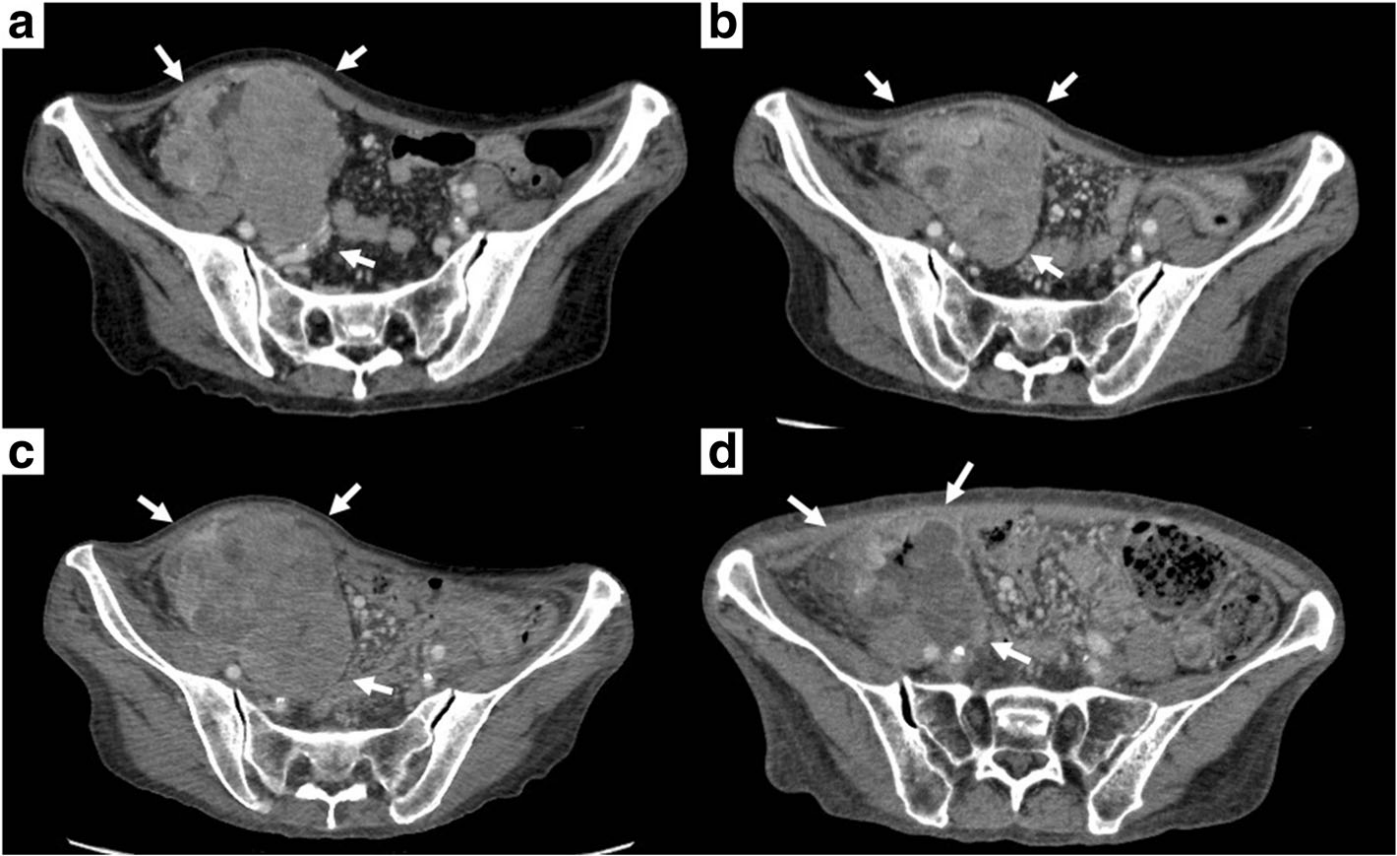
Computed tomography images. **a** Before treatment, 8.6-cm-diameter tumor infiltrating the abdominal wall, small intestine, and retroperitoneum. **b** After four cycles of chemotherapy, the tumor slightly shrank to 6.9 cm in diameter. **c** After eight cycles of chemotherapy, the tumor enlarged to 10 cm in diameter. **d** After radiotherapy, the tumor reduced to 6.6 cm in diameter with intratumor liquefaction degeneration

A 65-year-old healthy Japanese woman presented with a chief complaint of malaise and hematochezia. The physical examination revealed a 10-cm-diameter hard mass at the right lower quadrant of the abdomen without tenderness or cutaneous involvement. Laboratory findings demonstrated severe anemia and elevated tumor markers (hemoglobin, 2.9 mg/dl; carcinoembryonic antigen, 10.8 ng/ml; carbohydrate antigen 19-9, 21.6 U/ml). A colonoscopy showed a circumferential neoplastic lesion at the ascending colon which did not allow the scope to pass through (Fig. 1a). The biopsy reported a moderately differentiated tubular adenocarcinoma (Fig. 1b). RAS mutation was not detected. A CT showed an 8.6-cm-diameter tumor at the ascending colon which seemed to infiltrate the abdominal wall, small intestine, and retroperitoneum (Fig. 2a). Regional lymphadenopathies and ascites were also observed, but apparent distant metastases were not. Based on these findings, we made a clinical diagnosis of locally advanced ascending colon cancer stages T4b, N2a, M0, and IIIC according to the TNM classification [6]. Considering the possible extensive invasion to the surrounding structures, we recommended initial neoadjuvant chemotherapy followed by radical resection of the tumor.

**Fig. 3:**
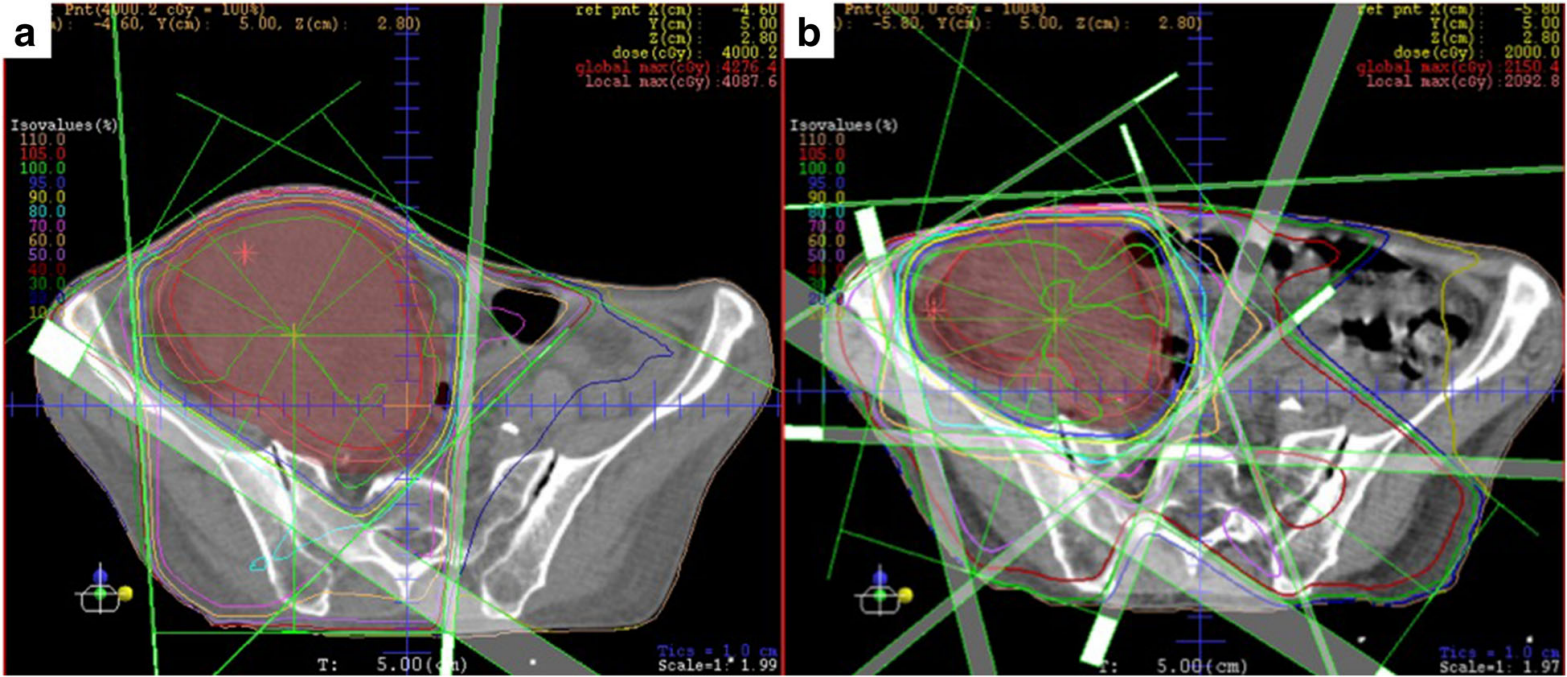
The image of radiation treatment planning system: an axial view of dose distribution. **a** We prescribed the initial dose of 40 Gy in 20 fractions with three-field technique. **b** After reconstituting the radiation field conforming with the shrunken tumor, we boosted the additional dose of 20 Gy in 10 fractions with five-field technique

**Fig. 4:**
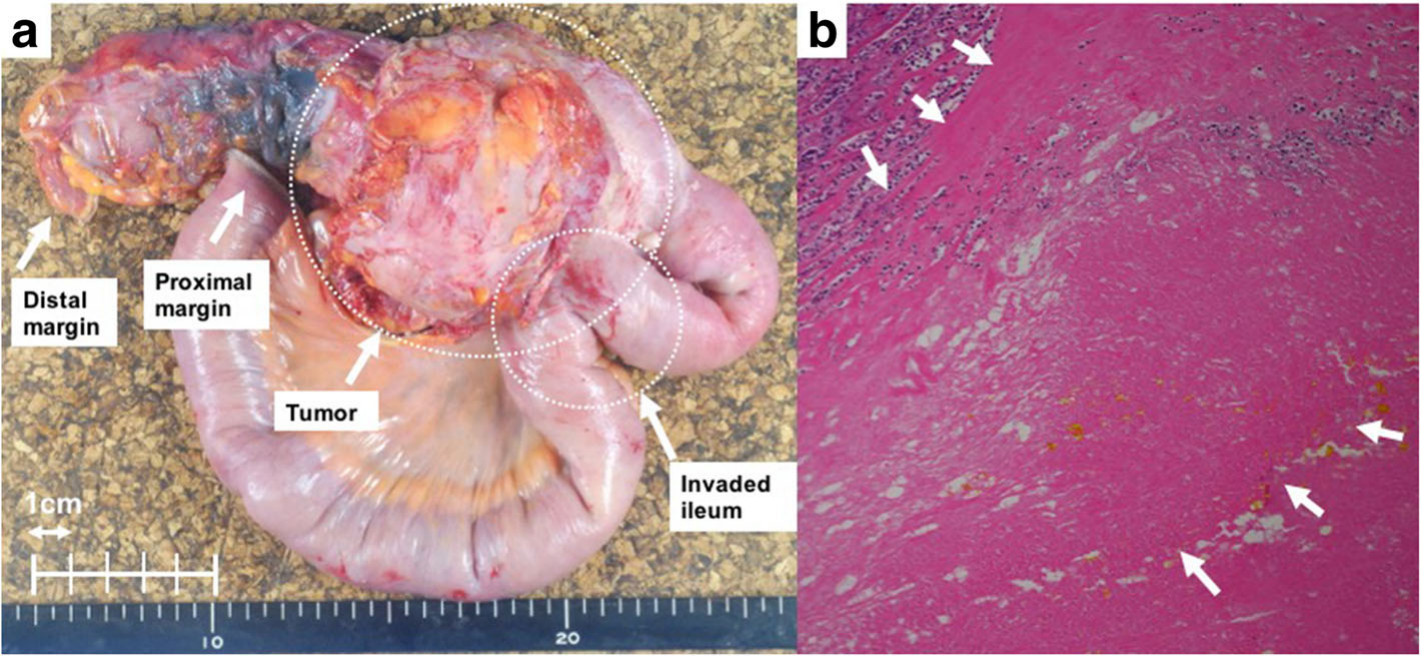
Macroscopic and microscopic images of resected specimen. **a** 9.0 × 7.0 cm tumor seemed to infiltrate the ileum. **b** Histopathologically, no residual cancer cells were found in the giant mass and marked stromal fibrosis replaced the tumor

The patient underwent 4 cycles of modified FOLFOX6 with panitumumab, and the tumor shrank only slightly to 6.9 cm in diameter (Fig. 2b). An additional 4 cycles of the same regimen were administered but the tumor actually enlarged to 10 cm in diameter (Fig. 2c), and thus, the disease was determined to be a progressive disease according to the Response Evaluation Criteria in Solid Tumors [7]. At this point, we felt more chemotherapies and/or surgeries were not recommended because of the evidence of disease progression and because the patient’s condition was quite frail. Instead, we recommended radiotherapy of 60 Gy in 30 fractions (Fig. 3). The patient tolerated the treatments well, and no serious adverse effects were reported. After the radiotherapy, the tumor shrank to 6.6 cm in diameter with intratumor liquefactive degeneration (Fig. 2d). One month after the radiotherapy, she underwent right hemicolectomy with D3 lymphadenectomy. Metastatic lesions of the liver or peritoneum were not observed. The right ovarian vessels and ileum at 10 cm proximal from the ileocecal valve were infiltrated by the tumor and resected concomitantly. The adjacent abdominal wall was intact. The transverse colon and ileum at 30 cm proximal from the ileocecal valve were cut and anastomosed. The macroscopic exam of the resected specimen showed a 9.0 × 7.0-cm circumferential tumor with a 4.5 × 3.5-cm ulcer at the ascending colon, which extensively penetrated the colon serosa and infiltrated the ileum and the ovarian vessels (Fig. 4a). Histopathologically, the primary tumor of the ascending colon and enlarged regional lymph nodes consisted in its totality of granuloma-like or fibrous tissues and no residual cancer cells were found (Fig. 4b). Pathological findings revealed complete response, and the final findings were ypT0, ypN0 (0/15), and ypStage0. Neoadjuvant treatment effect was grade 0 according to American Joint Committee on Cancer System [8]. The postoperative course was uneventful. The patient remains alive without any additional therapies after 24 months for follow-up, with no signs of recurrence.

## Discussions

The NCCN guideline describes that radiotherapy for colon cancer may be considered for very selected patients with disease characterized as T4 tumors penetrating to a fixed structure or for patients with recurrent disease [9]. The Japanese guideline for the treatment for colorectal cancer regards radiotherapy for colon cancer as palliative care (for the relief of symptoms) [4]. Thus, the oncological effectiveness of radiotherapy for colon cancer is considered to be limited. To our knowledge, this is the first report of preoperative radiotherapy leading to pathological complete response of a locally advanced colon cancer. The present case suggests that radiotherapy could yield great oncological benefits to patients with locally advanced colon cancer.

Although chemoradiotherapy for colon cancer rarely leads to pathological complete response, three studies have reported such outcome in the last 5 years (Table 1). They reported that preoperative chemoradiotherapy for locally advanced colon cancer might achieve R0 resection (91–100%) and occasionally, pathological complete response (3–38%) [10–12]. Although these studies involved a limited number of patients (21–34 patients) and short median follow-up periods (23–42 months), they described more favorable oncological outcomes compared to the previous studies reported over 10 years ago [13, 14]. It suggests that chemoradiotherapy for locally advanced colon cancer has been more refined and thus possibly exerts more beneficial oncological effects.

**Table 1:** Previous studies of pathological complete response of locally advanced colon cancer after neoadjuvant chemoradiotherapy

Reference	Inclusion period	No. of participants	Location of cancer	Regimen of chemotherapy	RT (Gy/Fr)	R0 (%)	pCR (%)	OS (%/year)	DFS (%/year)
Huang [10], Taiwan	2012–2016	36	Any part of colon	FOLFOX	45–50.4/25–28	31/34 (91.2%)	9/34 (26.4%)	88.7%/2	73.6%/2
Qiu [11], China	2010–2012	21	Sigmoid colon	Capecitabine based	46–50/23–25	20/21 (95.2%)	8/21 (38.1%)	100%/3	88.9%/3
Cukier [12], Canada	2000–2009	33	Any part of colon	5-FU based	36–50.4/18–28	33/33 (100%)	1/33 (3.0%)	85.9%/3	73.7%/3

*RT* radiotherapy, *pCR* pathological complete response, *OS* overall survival, *DFS* disease-free survival, *FOLFOX* folinic acid, 5-fluorouracil and oxaliplatin, *5-FU* 5-fluorouracil

The present case differs from the cases treated by chemoradiotherapy in that pathological complete response was achieved by radiotherapy after the failure of chemotherapy. Although there is a possibility that the chemotherapy might have contributed to the complete response, the choronological change in tumor size in relation to the treatments suggested that radiotherapy was much more effective than chemotherapy. We can list two possible reasons why the radiotherapy was effective. First, the tumor was giant, suspected to infiltrate the surrounding structures, and thus fixed on the right side of the abdomen, which could enhance the cell-killing effect of the radiation. Second, our patient underwent radiation at 60 Gy corresponding to a maximum dose according to the Japanese guideline for the treatment of colorectal cancer [4], which could be also effective. We could not continue chemotherapy with radiotherapy simultaneously; thus, we set the radiation dose to the maximum recommended. Although the patient received the high-dose radiation uneventfully, we should be observant of possible late complications because the tolerance dose of the colon leading to a 5% complication rate at 5 years is estimated to be between 45 and 55 Gy [15].

The appropriate interval between preoperative radiotherapy and surgery for colon cancer remains to be standardized, although 6–8 weeks has been the current standard interval between preoperative chemoradiotherapy and surgery for rectal cancer [16]. Some reports suggested that lengthening the interval improved the rate of pathological complete response [17]. Further studies are needed to determine the appropriate time to surgery.

We should also consider radiation exposure to the adjacent organs, especially the small bowel. Qin et al. reported that the incidence of postoperative anastomotic leak and stricture was higher in patients with locally advanced rectal cancer who underwent preoperative chemoradiotherapy than in patients who underwent preoperative chemotherapy without radiotherapy [18]. It is conceivable that using small bowel exposed to radiation for anastomosis contributes to the higher incidence of postoperative complications in the patients with colon cancer. In the present case, we tried to avoid such postoperative complications by resecting the ileum at 30 cm proximal to the ileocecal valve which was suspected to be intact from radiation.

## Conclusions

We report a first case of locally advanced colon cancer with pathological complete response after preoperative radiotherapy. The present case suggests that preoperative radiotherapy might be an effective treatment options for locally advanced colon cancer.

## Abbreviations

5-FU: 5-Fluorouracil
CT: Computed tomography
DFS: Disease-free survival
FOLFOX: Folinic acid, 5-fluorouracil and oxaliplatin
NCCN: National Comprehensive Cancer Network
OS: Overall survival
pCR: Pathological complete response
RT: Radiotherapy
